# Choking to Death

**Published:** 1858-02

**Authors:** 


### CHOKING TO DEATH,

FROM eating too fast or carelessly, is no unusual occurrence. A lady swallowed a triangular piece of chicken bone, while taking some soup; it lodged edgeways in the gullet, but no hold could be taken of it. For five days she could swallow nothing. Dr. DAVID RICE, of Leverett, Mass., was called, and attached to a whalebone as large a piece of dry sponge as he could push past the bone, then gradually filled the sponge with water, which caused it to swell and fill the whole cavity below; the sponge had only to be drawn up by the whalebone, bringing the bone with it.

This incident strikingly illustrates the nature of the practice of a scientific physician. He is often called on to give relief, in a combination of circumstances which never happened before, and he is required to act without time for consultation—is thrown instantaneously and wholly on his own resources, and with as triumphal results as in the case of Dr. Rice. An empiric, when his accustomed plan fails, is at his wits' end, and is utterly powerless. Let this be a warning to all our readers, never to send for any other than an educated physician, in any emergency, for with such only are you in safe and efficient hands; and if relief is possible, he can give it.

